# Supplementary material for: Educational Simulation Program Based on Korean Triage and Acuity Scale
Source: Int J Environ Res Public Health. 2020 Dec 3;17(23):9018. doi: 10.3390/ijerph17239018 (PMC7730408; doi:10.3390/ijerph17239018)
Supplement: Supplementary file 1 [file ijerph-17-09018-s001.pdf]

Simulation Program Scenario based on Korean Triage and Acuity Scale:

Table S1. Patient case overview.

**Symptom**	Epigastric pain	**Diagnosis**	Epigastric pain
**Patient’s name**	○ ○ ○		
**Patient characteristics**	Age: 37 years old	Gender: Male	
	Height: 181 cm	Weight: 75 kg	
**Class operation**	Running time: 10 min	Debriefing time: 20 min	
**Evaluation target**	Korean Triage and Acuity Scale<br>(KTAS) manager		
**Evaluation goal**	1. Ability to perform severity classification<br>2. Patient assessment ability<br>3. Ability to communicate therapeutically with the patient		
**Learning objectives**	1. Can perform severity classification quickly and accurately<br>2. Patient can be assessed according to their chief complaint<br>3. Can communicate therapeutically with the patient		
**Preparation facilities and equipment**	☐ Standardized patient’s casual clothes<br>(sports clothes top and bottom, hat, slippers)<br>☐ Computer<br>☐ Writing equipment<br>☐ Numeric rating scale, NRS<br>☐ Sphygmomanometer<br>☐ Glucometer<br>☐ Thermometer<br>☐ Desk, two chairs<br>☐ Patient name band<br>☐ A memo measuring the patient's vital signs		

Table 2. Situation introduction and guidelines for nurses.

**Patient’s name**	○ ○ ○
**Vital signs**	BP: 140/90, P: 75, RR: 25, T: 36.9 ℃, SpO2: 97%
**Patient’s age**	37 years old
**History**	Repeated hospitalization and discharge from the gastroenterology department of our hospital due to chronic pancreatitis.<br>Frequent visits to the emergency medical center due to alcohol use and abuse, and non-cooperative.<br>He visited the hospital for abdominal pain and requested the administration of narcotic analgesics.
**Instructions for nurses**	You are a nurse at OO Hospital Emergency Medical Center, and you are working as a KTAS classifier in the evening.<br>○ ○ ○ is a 37-year-old male who has been advised to stop drinking because of chronic pancreatitis and has repeatedly been admitted to and discharged from the gastroenterology department at our hospital.<br>He visits emergency medical centers mainly because of alcohol, and he shows abusive and non-cooperative attitudes toward medical staff, and continues to demand narcotic pain relievers for abdominal pain.<br>Even today, he is in a drunk state and says he has a pain in his stomach that is different from usual, and he is still in a situation where he asks for narcotic pain relievers in an uncooperative manner.<br>Assess patient condition and conduct KTAS classification.
**Important attention**	None
**Class operation**	Running time: 10 min
**Other**	As the scenario progresses, when checking vital signs, the patient provides a memo of the results of the vital signs.

Table 3. Debriefing.

Step	Questions
**Description phase**	This is to analyze your own simulation practice.<br>Questions were asked to talk about the situation, what the patient experienced, and what was difficult.
**Analysis phase**	This is about the experience or self-evaluation of the simulation practice.<br>Have you ever experienced a similar situation to the one today?<br>What kind of care did you perform well?<br>What do you regret?<br>What did you do when the patient complained of discomfort, and why?
**Application phase**	This step illustrates through simulation practice what nurses experience in clinical situations.<br>How can you apply what you have learned today in real clinical practice?<br>What is the most important thing you have learned from the simulation today?
